# Sensitivity to temporal fine structure and hearing-aid outcomes in older adults

**DOI:** 10.3389/fnins.2014.00007

**Published:** 2014-02-05

**Authors:** Elvira Perez, Abby McCormack, Barrie A. Edmonds

**Affiliations:** NIHR Nottingham Hearing Biomedical Research Unit, School of Medicine, University of NottinghamNottingham, UK

**Keywords:** lateralization, interaural phase difference, audiology, older adults, hearing aids

## Abstract

**Objective:** To investigate the effect of sensitivity to temporal fine structure (TFS) on subjective measures of hearing aid outcome.

**Design:** Prior to receiving hearing aids, participants completed a test to assess sensitivity to TFS and two self-assessment questionnaires; the Glasgow Hearing Aid Benefit Profile (GHABP), and the Speech, Spatial and Qualities of hearing (SSQ-A). Follow-up appointments, comprised three self-assessment questionnaires; the GHABP, the SSQ-B, and the International Outcome Inventory for Hearing Aid Outcomes (IOI-HA).

**Study sample:** 75 adults were recruited from direct referral clinics.

**Results:** Two thirds of participants were found to have good sensitivity to TFS; listeners with good sensitivity to TFS rated their hearing abilities higher at pre-fitting (SSQ-A) than those with poor sensitivity to TFS. At follow-up, participants with good sensitivity to TFS showed a smaller improvement on SSQ-B over listeners with poor sensitivity to TFS. Among the questionnaires, only the SSQ showed greater sensitivity to measure subjective differences between listeners with good and poor sensitivity to TFS.

**Conclusions:** The clinical identification of a patient's ability to process TFS information at an early stage in the treatment pathway could prove useful in managing expectations about hearing aid outcomes.

## Introduction

Presbycusis is characterized by gently-sloping high-frequency hearing loss. It is often first revealed to the listener through a reduced understanding of conversational speech, particularly when there is a source of background noise. A common treatment for presbycusis is provision of hearing aids. Hearing aids make understanding speech much easier for the vast majority of people in a range of situations. However, listening in complex or noisy environments can remain challenging for some people even after provision of amplification (Moore et al., 1999). As the ability to understand speech is only moderately associated with audiometric threshold (Ching et al., 1998), factors other than reduced audibility may contribute to the communication difficulties experienced by some patients. For example, when competing sound sources are spatially separated, spatial hearing plays an important role for speech intelligibility. It is well established that certain acoustical and perceptual mechanisms can lead to large speech intelligibility improvements (e.g., Zurek, 1993; Freyman et al., 1999). However, listeners have to have access to spatially salient acoustic cues to be able to take advantage of those mechanisms. Previous research showed that spatial hearing is mediated by various types of binaural acoustic cues: interaural time differences (ITDs), which, for on-going tones, translate to interaural phase differences (IPDs), interaural level differences (ILDs), and monaural spectral cues (see Blauert, 1983 for a review). ITDs arise as a result of the physical separation of a listener's ears and provide information about the left-right position of a sound source. ITDs are perceptually most potent below about 1–0.75 kHz and there is evidence regarding neural firing tracking the phase of a signal up to about 1.5 kHz, (Neher et al., 2009 for a review). Registration of IPDs reflect fine structure coding and are presumed to involve the comparison of phase-locked inputs in the two ears, a process that forms the basis of the coincidence detection model of binaural hearing (Jeffress, 1948). Consequently, IPDs provide an accepted metric for neural synchrony and sensitivity to temporal fine structure (TFS).

TFS information is useful (for normal-hearing listeners at least) for frequencies lower than 1000 Hz because TFS information is thought to be important for the perception of F0 information (Moore et al., 1984; Hartmann and Doty, 1996), and for the discrimination of IPDs (Hafter et al., 1979). In this study we have manipulated the IPD of the waveform fine structure for measuring the ITDs of periodic inputs such as pure tones. The TFS test is based on measuring thresholds for detecting an IPD for pure tones, where there is an interaural disparity in the TFS only. Listeners must be sensitive to TFS to detect such a disparity, which is usually heard as a shift in the position of the tone inside the head (Hopkins and Moore, 2010a,b).

It has been suggested that if the amount of TFS information in a speech signal is varied then listeners with sensori-neural hearing loss find the speech less intelligible than normally-hearing listeners (Lorenzi et al., 2006; Hopkins et al., 2008; Hopkins and Moore, 2010a,b). When sensitivity to TFS information is measured with tonal stimuli the relationship with speech intelligibility measures is not so clear. Hopkins and Moore (2011) found that after controlling for hearing loss, sensitivity to monaural TFS was correlated with speech reception thresholds (SRTs), but not sensitivity to binaural TFS cues. Strelcyk and Dau (2009), on the other hand, found that sensitivity to TFS was associated with SRTs against a multi-talker background, but not against an amplitude-modulated noise masker. Nonetheless, it is thought that the ability to exploit TFS information is poorer in adults with sensori-neural loss than normally-hearing listeners (Lacher-Fougere and Demany, 1998; Moore and Skrodzka, 2002; Hopkins et al., 2008; Strelcyk and Dau, 2009; Ardoint et al., 2010; Hopkins and Moore, 2010a,b), and varies among listeners with similar audiometric configurations (Hopkins et al., 2008; Strelcyk and Dau, 2009; Hopkins and Moore, 2010a,b). Consequently, it is thought that sensitivity to TFS information could account for some of the variability observed in the amount of benefit that patients report to receive from hearing aids. However, to our knowledge there is no evidence in the literature describing the effect of reduced sensitivity to TFS information on actual hearing-aid outcomes, as might be determined in the clinic.

The aim of this study was to investigate the effect of sensitivity to TFS information on hearing-aid outcomes. In an earlier report (Perez et al., 2012), a group of presbycusic participants completed tests of sensitivity to TFS information, temporal resolution (gap detection) and frequency resolution (notched-noise). We found that sensitivity to TFS information appeared to contribute to the degree of difficulty these participants reported experiencing on self-report questionnaires prior to the fitting of their hearing aids (portions of this data are also reported here for ease of reading). In the current report, we followed these patients for a period of 6 months after their hearing aid fittings to determine whether listeners with good sensitivity also perform better on hearing-aid outcomes. We hypothesized that those listeners with good sensitivity to TFS information would experience better hearing-aid outcomes than those with impaired TFS processing abilities.

## Methods

### Procedure

The recruitment of participants was made via leaflets distributed to patients attending Nottingham Audiological Services for a direct referral assessment. All participants that enrolled in the study met the following selection criteria: (a) followed General Practice (GP) direct-referral route to audiology, (b) 50+ years of age, (c) bilaterally symmetrical sensori-neural hearing loss, (d) had not previously worn a hearing aid, (e) normal or corrected-to-normal vision. Our sample comprised 75 adults (44 men and 31 women) with a mean age of 72.24 ± 0.82 (range age 51–85 years) with mild-to-moderate hearing loss. Ethical approval for this study was obtained from the Derbyshire Research Ethics Committee.

Participants were tested by a member of the research team on three occasions. The first testing session took place prior to the patient being fitted with a hearing aid. During this session, participants completed a short test to determine their sensitivity to TFS and a number of self-report assessment questionnaires. The second and third research appointments took place 3- and 6-month post-hearing-aid fitting, in which participants completed a number of self-report outcome questionnaires.

### Self-report questionnaires

In order to ascertain the degree of difficulty experienced prior to provision of a hearing aid in a range of listening scenarios, all participants were asked to complete the first part of the Glasgow Hearing Aid Benefit Profile (GHABP: Gatehouse, 1999) and the Speech, Spatial and Qualities of Hearing (SSQ-A: Gatehouse and Noble, 2004) questionnaires during the first testing session. Part one of the GHABP asks participants to rate themselves using a 5-point ordinal scale on two dimensions: Initial Disability and Handicap on four pre-specified listening circumstances which may commonly occur in the lives of people with hearing loss, (e.g., “*Listening to the television with other family or friends when the volume is adjusted to suit other people*”) and four self-nominated listening scenarios which allows the listener to specify additional listening circumstances of importance and relevance to their everyday communication circumstances (e.g., “*Listening to music in a concert hall*.”). Higher ratings on each of these dimensions indicate greater levels of difficulty or worry. The SSQ-A asks participants to rate their listening abilities using an ordinal scale (0–10) on three sub-scales: Speech, Spatial, and Qualities on 14, 17, and 18 pre-specified listening scenarios respectively (e.g., Speech sub-scale: “you are talking with one other person and there is a TV on in the same room. Without turning the TV down, can you follow what the person you're talking to says?”) Higher ratings on the SSQ-A indicate greater levels of perceived ability.

In order to ascertain the degree of benefit experienced after receiving a hearing aid, participants were asked to complete the second part of the GHABP, the SSQ-B (Jensen et al., 2009), and the International Outcome Inventory for Hearing Aids (IOI-HA, Cox and Alexander, 2002). The second part of the GHABP uses four pre-defined subscales for monitoring hearing-aid outcomes: Usage, Benefit, Residual Disability (difficulties still present while using the hearing aid), and Satisfaction. The SSQ-B is very similar to the SSQ-A, but asks participants to compare their hearing abilities now (aided) with their abilities prior to provision of a hearing aid on an ordinal scale ranging from −5 (much worse) to +5 (much better). The IOI-HA questionnaire uses a 5-point nominal scale (e.g., “helped not at all” through to “helped very much”) to record self-report scores for seven outcome dimensions: Use, Benefit, Residual Activity Limitation (difficulties still present while using the hearing aid that affect the users day-to-day activities), Satisfaction, Residual Participation Restriction (difficulties still present while using the hearing aid that affect the users social interactions), Impact on Others, and Quality of Life. For example, Residual Activity Limitation is assessed with the following question: “Think again about the situation where you most wanted to hear better. When you use your present hearing aid(s), how much difficulty do you STILL have in that situation?”

These questionnaires can be accessed online at: http://www.ihr.mrc.ac.uk/products/display/questionnaires http://www.harlmemphis.org/index.php/clinical-applications/ioi-ha/

### Hearing assessments

#### Hearing thresholds/0.25–8 kHz

Air-conduction audiometry without masking was used to calculate hearing thresholds at 0.25, 0.5, 1, 2, 4, and 8 kHz in accordance with the British Society of Audiology (BSA) guidelines (2011) by a qualified audiologist as part of the routine direct referral assessment process using a Siemens Unity 1 or 2 audiometers with TDH39 headphones. Air-conduction audiometry without masking consists on measuring the quietest percept of a sound (target tone). Participants are asked to press a button as soon as they hear a tone and keep it pressed for as long as they hear the tone, no matter which ear they hear it in. Participants are asked to release the button as soon as they no longer hear the tone. According the BSA guidelines, the professional administrating the audiometry should start presenting the tones at the better-hearing ear (according to the subject's account) and at 1000 Hz. Next, test 2000, 4000, 8000, 500, and 250 Hz in that order. It is also recommended to vary the length of the tone presentation to ensure that the timing of each tone is not predictable.

#### TFS/0.5 kHz

Sensitivity to TFS was measured using the TFS-LF method (Hopkins and Moore, 2010a,b) over Sennheiser HD-25. The task utilizes a two-interval two-alternative forced choice (2I-2AFC) task. Each interval contained four 0.5 kHz pure tones in either *AAAA* or *ABAB* sequences. In *AAAA* intervals, all the tones were presented diotically. In *ABAB* intervals, the first and third tones were diotic whilst the 2nd and 4th tones were presented with an IPD (ΔØ). Participants were asked to identify which interval contained the tones that appeared to change in location. A two-up, one-down adaptive procedure was used to vary ΔØ. At the beginning of a run ΔØ was set to a maximum value of 180°. Thresholds were calculated by measuring the geometric mean of ΔØ at the last six turn points which corresponded to the 71% correct point. However, the adaptive procedure terminated early if this maximum value was reached twice before the second turn point, or at all after the second turn point. In this situation, the program reverted to a non-adaptive (method of constant measures) procedure in which a further 40 trials were presented with ΔØ fixed at its maximum value and a percentage correct score was calculated. Discriminability index (d′) values for the TFS test were calculated using a table of d′ values for two-alternative forced choice procedures (Hacker and Ratcliff, 1979) which was 0.78. For Thresholds measured using the percent-correct procedure we followed Hopkins and Moore (2010a,b) approach by linearly extrapolating the threshold value of ΔØ needed for 71% correct from the d′ scores, so that results from percent-correct and adaptive procedures could be compared.

$$\begin{array}{l} (\Delta \emptyset \text{extrapolated}=(0.78\times 180^{{}^{\circ}})/\text{d}\prime \text{ from percent }\\ \text{ correct procedure}) \end{array}$$

All participants concluded a practice run to ensure they understood the task. Signals used in the measurement of TFS were presented with a sampling rate of 44.1 kHz, using a PC and an external sound card (ECHO Gina 3D).

All hearing assessments were conducted in a double-walled, sound proof booth

### Statistical methodology

The statistical methodology employed in this study includes basic descriptive analysis, *post-hoc* paired-sample *t*-tests, One-Way ANOVA and Pearson correlations. Calculation of discriminability index (d′) values for the TFS is described in section Hearing assessments, TFS/0.5 kHz.

## Results

### Pre-fitting assessments

**Figure 1:**
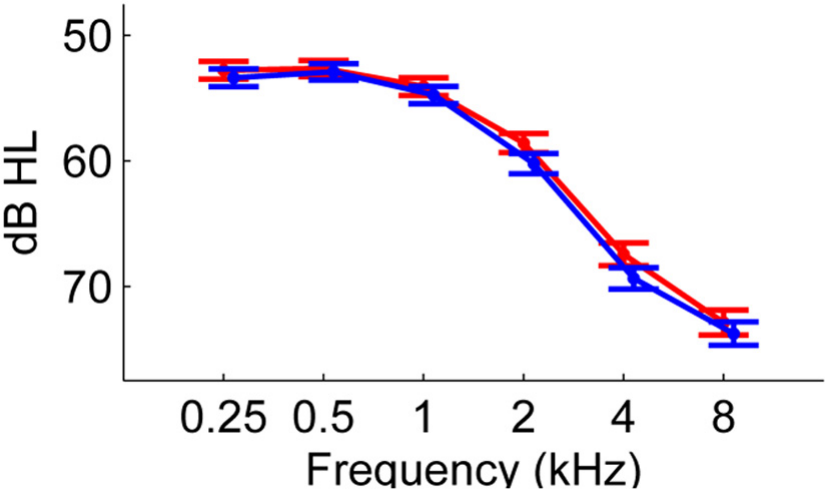
**Mean air-conduction PTA thresholds of 75 participants measured for right (red circles) and left (blue circles) ears**. Error bars show ±1 standard error of the mean.

Although hearing thresholds were classified to be bilaterally symmetric, the six-frequency pure-tone average hearing thresholds measured at the better ear and the poorer ear (34.5 ± 1.04 dB HL at the better ear) were found to be significantly different [*t*_(1, 74)_ = −9.12, *p* < 0.001]. Audiogram for left and right is shown in Figure 1.

In the following sections, we describe how age and hearing loss related to the self-reported assessments of hearing difficulty measured on the GHABP and SSQ-A. Whilst it was anticipated that hearing loss would account for most of the variability observed in hearing difficulties reported, we hypothesized that some of the variability observed in the difficulties experienced by these patients might be explained by their sensitivity to TFS information.

#### Glasgow hearing aid benefit profile (GHABP): assessment questionnaire

**Figure 2:**
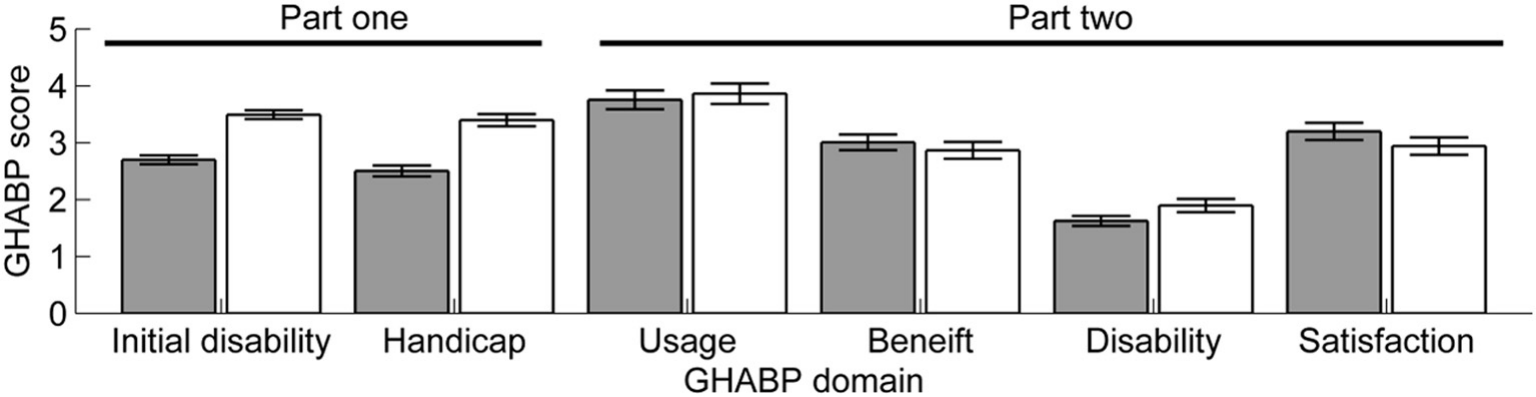
**Mean GHABP scores for pre-defined (gray bars) and self-nominated (white bars) scenarios**. Error bars show ±1 standard error of the mean.

Participants reported experiencing “Great difficulty” (3.06 ± 0.57) on the Initial Disability sub-scale and “Moderate” levels of worry on the Handicap sub-scale (2.9 ± 0.78). Initial Disability and Handicap scores were strongly correlated with one another, but were not correlated with age or audiometric threshold (see Table 1). In addition to the four pre-defined listening scenarios described in the GHABP, participants had the option of nominating an additional four listening scenarios. All participants completed the four pre-defined listening scenarios and 26 provided self-nominated scenarios: five participants self-nominated a single additional scenario, six participants provided two self-nominated scenarios, seven participants provided three self-nominated scenarios, and eight participants provided four self-nominated scenarios. For those participants who provided self-nominated scenarios, the four pre-defined listening situations (S1–S4) were scored as significantly less difficult [Initial Disability: *t*_(1, 28)_ = −7.72, *p* < 0.01] and less worrying [Handicap: *t*_(1, 28)_ = −7.95, *p* < 0.01] than the self-nominated listening situations (See Figure 2).

**Table 1:** **Results of Pearson correlation for pre-fitting assessment GHABP**.

	**1**	**2**	**3**	**4**
1. Initial disability	−	*r* = 0.8^**^	*r* = 0.2	*r* = 0.1
2. Handicap	*r* = 0.8^**^	−	*r* = 0.2	*r* = 0.1
3. Age	*r* = 0.2	*r* = 0.2	−	*r* = 0.4^**^
4. Audiometry	*r* = 0.1	*r* = 0.1	*r* = 0.4^**^	−

Notes:

** *Correlation is significant at 0.01 (2-tailed)*.

#### Speech, spatial and qualities of hearing: assessment (SSQ-A)

**Figure 3:**
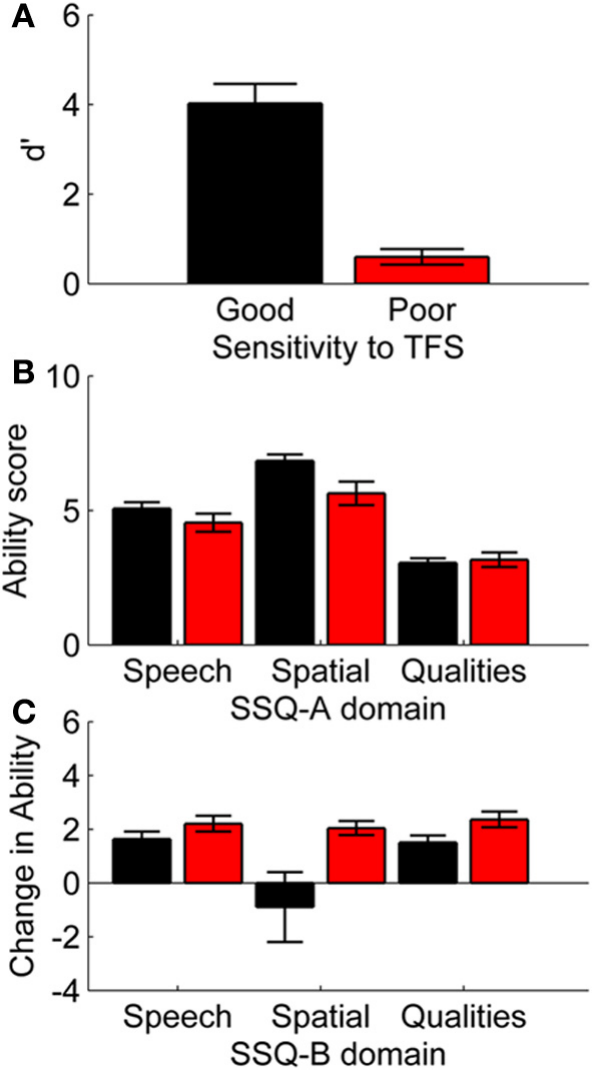
**Sensitivity to TFS information and self-reported listening abilities. (A)** discriminability index (d′) of participants classified as having good or poor sensitivity on the basis of whether they completed the TFS-LF test adaptively (good sensitivity) or reverted to a method of constant measures (poor sensitivity), **(B)** SSQ-A self-report scores for participants with good sensitivity to TFS information (black bars) or poor sensitivity to TFS information (red bars), **(C)** SSQ-B self-report scores for participants with good sensitivity to TFS information (black bars) or poor sensitivity to TFS information (red bars). Error bars show ±1 standard error of the mean.

The majority of participants reported moderate levels of hearing ability on the Speech, Spatial and Qualities sub-scales (see Figure 3). Correlations are described in Table 2.

**Table 2:** **Results of pearson correlation for pre-fitting assessment SSQ-A and TFS**.

		**1**	**2**	**3**	**4**	**5**	**6**	**7**	**8**
1.	Speech	−	*r* = 0.7^**^	*r* = 0.7^**^	*r*= −0.3^*^	*r*= −0.3^**^	*r*= −0.3^**^	*r* = 0.1	*r* = 0.1
2.	Spatial	*r* = 0.7^**^	−	*r* = 0.7^**^	*r*= −0.3^*^	*r*= −0.3^**^	*r*= −0.3^**^	*r* = 0.3^*^	*r* = 0.1
3.	Qualities	*r* = 0.7^**^	*r* = 0.7^**^	−	*r*= −0.1	*r*= −0.3^**^	*r*= −0.3^**^	*r* = 0.3^*^	*r* = 0.1
4.	Audiometry	*r*= −0.3^*^	*r*= −0.3^*^	*r*= −0.1	−	*r* = 0.1	*r* = 0.1	*r*= −0.1	*r* = 0.4^**^
5.	Initial disability	*r*= −0.3^**^	*r*= −0.3^**^	*r*= −0.3^**^	*r* = 0.1	−	*r* = 0.8^**^	*r* = 0.1	*r* = 0.2
6.	Handicap	*r*= −0.3^**^	*r*= −0.3^**^	*r*= −0.3^**^	*r* = 0.1	*r* = 0.8^**^	−	*r* = 0.1	*r* = 0.2
7.	TFS (d′)	*r* = 0.1	*r* = 0.3^*^	*r* = 0.3^*^	*r*= −0.1	*r* = 0.1	*r* = 0.1	−	*r*= −0.27^*^
8.	Age	*r* = 0.1	*r* = 0.1	*r* = 0.1	*r* = 0.4^**^	*r* = 0.2	*r* = 0.2	*r*= −0.27^*^	−

Notes:

* Correlation is significant at 0.05 level (2-tailed);

** *correlation is significant at 0.01 (2-tailed)*.

### Sensitivity to temporal fine structure (TFS)

Altogether, 49 participants completed the TFS-LF task using the adaptive procedure while 26 participants reverted to the method of constant measures (i.e., discriminating tones with a fixed phase shift of 180°). Sensitivity to TFS information was confirmed by comparing discriminability index (d′) values for the two groups (see Figure 3A). The participants that completed the adaptive version of the test were found to have significantly greater sensitivity to TFS information than those listeners who reverted to the constant measures version of the test [*F*_(1, 74)_ = 31.43, *p* < 0.01].

Sensitivity to TFS (d′) was weakly associated with age, and moderately associated with self-report scores of the Spatial and Quality sub-scales of the SSQ-A (see Table 2). Participants with good sensitivity to TFS reported significantly greater confidence in their Spatial processing abilities [*F*_(1, 73)_ = 7.23, *p* < 0.01] than participants with poorer sensitivity to TFS (see Figure 3B).

### Hearing aid outcomes at the 3-month follow-up

#### Glasgow hearing aid benefit profile (GHABP): outcome questionnaire

The mean GHABP part two self-report scores for Usage, Benefit, Residual Disability, and Satisfaction are shown in Figure 2. For those listeners who provided self-nominated listening scenarios, there were significant differences between pre-defined and self-nominated scenario scores for Benefit [*t*_(1, 26)_ = 2.89, *p* = 0.08], Residual Disability [*t*_(1, 26)_ = −2.22, *p* = 0.035], and Satisfaction [*t*_(1, 26)_ = 2.99, *p* = 0.006].

There was no association between GHABP self-reported outcomes and severity of hearing loss, or sensitivity to TFS information. Significant associations are described in Table 3.

**Table 3:** **Results of pearson correlation for GHABP 3-month follow-up**.

		**1**	**2**	**3**	**4**	**5**	**6**	**7**
1.	Usage	−	*r* = 0.5^**^	*r* = 0.5^**^	*r* = 0.1	*r* = 0.3^*^	*r* = 0.3^*^	*r* = −0.36^*^
2.	Benefit	*r* = 0.5^**^	−	*r* = 0.5^**^	*r* = −0.4^**^	*r* = −0.1	*r* = −0.1	*r* = 0.2
3.	Satisfaction	*r* = 0.5^**^	*r* = 0.5^**^	−	*r* = −0.4^**^	*r* = −0.1	*r* = −0.1	*r* = 0.2
4.	Residual disability	*r* = 0.1	*r* = −0.4^**^	*r* = −0.4^**^	−	*r* = 0.1	*r* = 0.1	*r* = 0.1
5.	Handicap	*r* = 0.3^*^	*r* = −0.2	*r* = −0.1	*r* = 0.1	−	*r* = 0.1	*r* = 0.2
6.	Initial disability	*r* = 0.3^*^	*r* = −0.2	*r* = −0.1	*r* = 0.1	*r* = −0.1	−	*r* = 0.2
7.	Age	*r* = −0.36^*^	*r* = 0.2	*r* = 0.2	*r* = 0.1	*r* = 0.2	*r* = 0.2	−

Notes:

* Correlation is significant at 0.05 level (2-tailed);

** *correlation is significant at 0.01 (2-tailed)*.

#### Speech, spatial and qualities: benefit (SSQ-B)

Participants reported moderate improvements in listening ability on all three sub-scales of the SSQ-B (Speech, 1.9; Spatial 1.3; Qualities 1.9). See Table 4 for associations between SSQ-B outcomes and other variables.

**Table 4:** **Results of pearson correlation for SSQ-B 3-month follow-up**.

	**Speech-B**	**Spatial-B**	**Qualities-B**
Speech-B	−	*r* = 0.7^**^	*r* = 0.7^**^
Spatial-B	*r* = 0.7^**^	-	*r* = 0.7^**^
Qualities-B	*r* = 0.7^**^	*r* = 0.7^**^	−
Speech-A	*r* = 0.5^**^	*r* = 0.5^**^	*r* = 0.5^**^
Spatial-A	*r* = 0.5^**^	*r* = 0.5^**^	*r* = 0.5^**^
Qualities-A	*r* = 0.5^**^	*r* = 0.5^**^	*r* = 0.5^**^
Usage	*r* = −0.4^**^	*r* = −0.4^**^	*r* = −0.4^**^
Benefit	*r* = −0.4^**^	*r* = −0.4^**^	*r* = −0.4^**^
Satisfaction	*r* = −0.4^**^	*r* = −0.4^**^	*r* = −0.4^**^
Residual disability	*r* = −0.3^**^	*r* = −0.3^**^	*r* = −0.3^**^
Hearing thresholds (0.25 kHz right ear)	*r* = 0.1	*r* = 0.3^*^	*r* = 0.1
Age	*r* = 0.1	*r* = 0.1	*r* = 0.1

Notes:

* Correlation is significant at 0.05 level (2-tailed);

** *correlation is significant at 0.01 (2-tailed)*.

Participants with poor sensitivity to TFS (constant-measures TFS group) reported experiencing greater levels of improvement on all three of the SSQ-B sub-scales. Ratings of improvement were significant different between the two groups on the Qualities [*F*_(1, 67)_ = 4.22, *p* < 0.05] sub-scale. It can be seen from Figure 3C that, on average, listeners with good sensitivity to TFS information reported a decrement in their spatial processing abilities following hearing aid provision.

#### International outcome inventory for hearing aids (IOI-HA)

Self-reported outcomes obtained on the seven questions of the IOI-HA were strongly associated with one another, however, usage did not correlate with Residual Activity Limitations or Residual Participation Restrictions; Satisfaction was not associated with Impact on Others. There were no associations with age, hearing loss (better ear average) or sensitivity to TFS. However, Impact on Others was associated with degree of hearing loss at 4 kHz for the left ear. See Table 5 for associations between IOI-HA dimensions, GHABP post-fitting sub-scales and SSQ-B outcomes.

**Table 5:** **Results of pearson correlation for IOI-HA 3-month follow-up**.

	**Usage**	**Benefit**	**RAL**	**Satisfaction**	**RPR**	**IoO**	**QL**
Usage	−	*r* = 0.5^**^	*r* = 0.2	*r* = 0.5^**^	*r* = 0.2	*r* = 0.4^**^	*r* = 0.4^**^
Benefit	*r* = 0.5^**^	−	*r* = 0.4^**^	*r* = 0.8^**^	*r* = 0.3^*^	*r* = 0.8^**^	*r* = 0.3^*^
*R*AL	*r* = 0.1	*r* = 0.4^**^	−	*r* = 0.3^*^	*r* = 0.3^*^	*r* = 0.3^*^	*r* = 0.3^*^
Satisfaction	*r* = 0.5^**^	*r* = 0.8^**^	*r* = 0.3^*^	−	*r* = 0.2^*^	*r* = 0.1	*r* = 0.2^*^
RPR	*r* = 0.1	*r* = 0.3^*^	*r* = 0.3^*^	*r* = 0.2^*^	−	*r* = 0.2^*^	*r* = 0.2^*^
IoO	*r* = 0.4^**^	*r* = 0.8^**^	*r* = 0.3^*^	*r* = 0.1	*r* = 0.2^*^	−	*r* = 0.2^*^
QL	*r* = 0.4^**^	*r* = 0.3^*^	*r* = 0.3^*^	*r* = 0.2^*^	*r* = 0.2^*^	*r* = 0.2^*^	−
Age	*r* = 0.1	*r* = 0.1	*r* = 0.1	*r* = 0.1	*r* = 0.1	*r* = 0.1	*r* = 0.1
Audiometry	*r* = 0.1	*r* = 0.1	*r* = 0.1	*r* = 0.1	*r* = 0.1	*r* = 0.1	*r* = 0.1
Hearing threshold (4 kHz left ear)	*r* = 0.1	*r* = 0.1	*r* = 0.1	*r* = 0.1	*r* = 0.1	*r* = 0.3^*^	*r* = 0.1
TFS (d′)	*r* = 0.1	*r* = 0.1	*r* = 0.1	*r* = 0.1	*r* = 0.1	*r* = 0.1	*r* = 0.1
Usage GHABP	*r* = 0.6^**^	*r* = 0.2^*^	*r* = 0.1	*r* = 0.1	*r* = 0.1	*r* = 0.3^*^	*r* = 0.3^*^
Benefit GHABP	*r* = 0.4^*^	*r* = 0.6^**^	*r* = 0.4^*^	*r* = 0.5^**^	*r* = 0.4^*^	*r* = 0.4^*^	*r* = 0.4^*^
Satisfaction GHABP	*r* = 0.4^*^	*r* = 0.3^*^	*r* = 0.4^*^	*r* = 0.6^**^	*r* = 0.4^*^	*r* = 0.5^*^	*r* = 0.4^*^
Residual disability GHABP	*r* = 0.1	*r* = 0.3^*^	*r* = 0.3^*^	*r* = 0.1	*r* = 0.1	*r* = 0.2^*^	*r* = 0.2^*^
Speech-B	*r* = 0.4^*^	*r* = 0.5^**^	*r* = 0.4^*^	*r* = 0.2^*^	*r* = 0.7^**^	*r* = 0.2^*^	*r* = 0.7^**^
Spatial-B	*r* = 0.5^**^	*r* = 0.4^*^	*r* = 0.5^**^	*r* = 0.1	*r* = 0.2^*^	*r* = 0.5^**^	*r* = 0.2^*^
Qualities-B	*r* = 0.7^**^	*r* = 0.2^*^	*r* = 0.2^*^	*r* = 0.1	*r* = 0.1	*r* = 0.2^*^	*r* = 0.7^**^

Notes:

* Correlation is significant at 0.05 level (2-tailed);

** *correlation is significant at 0.01 (2-tailed). RAL, Residual Activity Limitation; RPR, Residual Participation Restriction; IoO, Impact on Others; and QL, Quality of Life*.

### Hearing-aid outcomes at the 6-month follow-up

Of the original sample of 75 people who participated at the 3-month follow-up, only 54 attended the 6-month follow-up appointment (72% retention rate). The association between age and GHABP Residual Disability was preserved at the 6-month follow-up (*r* = −0.36, *p* ≤ 0.05) which suggests that this relationship is fairly stable. The association first observed at the 3-month follow-up between low-frequency hearing loss and self-reported outcome was also observed at the 6-month follow-up appointment. Thus although, presbycusis is generally accepted to reflect high-frequency loss, consideration of low-frequency audiometric configurations appears to be important to self-reported outcomes. Table 6 provides a summary of some of the key findings from this visit.

**Table 6:** **Results of pearson correlation for 6-month follow-up outcomes with degree if hearing loss**.

	**Hearing thresholds (0.25 kHz)**	**Hearing thresholds (0.5 kHz)**	**Hearing thresholds (1 kHz)**	**Hearing thresholds (2 kHz)**
Usage GHABP	*r* = 0.2	*r* = 0.2	*r* = 0.2	*r* = 0.1
Benefit GHABP	*r* = 0.3^*^	*r* = 0.3^*^	*r* = 0.2	*r* = 0.1
Satisfaction GHABP	*r* = 0.1	*r* = 0.3^*^	*r* = 0.1	*r* = 0.1
Residual disability GHABP	*r* = 0.1	*r* = 0.2	*r* = 0.1	*r* = 0.1
Speech-B	*r* = 0.3^*^	*r* = 0.3^*^	*r* = 0.1	*r* = 0.1
Spatial-B	*r* = 0.3^*^	*r* = 0.3^*^	*r* = 0.1	*r* = 0.1
Qualities-B	*r* = 0.1	*r* = 0.3^*^	*r* = 0.2	*r* = 0.2
Usage IOI-HA	*r* = 0.1	*r* = 0.1	*r* = 0.2	*r* = 0.2
Benefit IOI-HA	*r* = 0.1	*r* = 0.1	*r* = 0.1	*r* = 0.2
RAL IOI-HA	*r* = 0.1	*r* = 0.1	*r* = 0.2	*r* = 0.2
Satisfaction IOI-HA	*r* = 0.3^*^	*r* = 0.3^*^	*r* = 0.2	*r* = 0.2
RPR IOI-HA	*r* = 0.1	*r* = 0.1	*r* = 0.2	*r* = 0.3^*^
IoO IOI-HA	*r* = 0.1	*r* = 0.1	*r* = 0.3^*^ (right ear only)	*r* = 0.3^*^
QL IOI-HA	*r* = 0.1	*r* = 0.1	*r* = 0.05	*r* = 0.1

Notes:

* *Correlation is significant at 0.05 level (2-tailed). RAL, Residual Activity Limitation; RPR, Residual Participation Restriction; IoO, Impact on Others; and QL, Quality of Life*.

Differences in outcome reported at the 3- and 6-month follow-up appointments were compared using *post-hoc* paired-sample *t*-tests for each of the self-report sub-scales. There were no significant improvements in outcome as measured on the GHABP or the SSQ-B over the 6-month follow-up period. However, IOI-HA Usage ratings increased at the 6-month follow-up [*t*_(1, 49)_ = −2.09, *p* < 0.05], and IOI-HA Residual Activity Limitations decreased during the same period [*t*_(1, 48)_ = −2.27, *p* < 0.05]. Participants with the poorest sensitivity to TFS continued to experience better outcomes (SSQ-B) at the 6-month follow-up than those with good sensitivity to TFS [Speech: *F*_(1, 48)_ = 5.38, *p* < 0.05; Qualities: *F*_(1, 48)_ = 4.36, *p* < 0.05].

## Discussion

In this observational case series, we monitored the auditory rehabilitation of 75 older adults for a period of 6-months following receipt of their first hearing aid. All patients received standard audiological management pathways (initial audiological assessment and provision of hearing aids) for sensori-neural hearing loss. No experimental interventions or treatment groups were used. However, patients did complete a non-standard pre-fitting assessment to determine sensitivity to TFS information, and a range of non-standard pre- and post-fitting self-report questionnaires. The main purpose of this study was to assess how sensitivity to TFS information contributed to the hearing difficulties that the group faced pre- and post-provision of hearing aids.

### Sensitivity to temporal fine structure

It is generally accepted that speech perception deteriorates with increasing age (Plomp and Mimpen, 1979; Duquesnoy, 1983; Dubno et al., 2002) and hearing loss (Houtgast and Festen, 2008). A number of studies have shown that listeners with sensori-neural hearing loss are less able to exploit TFS cues for speech understanding than normally-hearing controls (Lorenzi et al., 2006; Hopkins et al., 2008; Ardoint et al., 2010; Hopkins and Moore, 2010a,b). However, there is no evidence to indicate that sensitivity to TFS information is dependent on the severity of hearing loss. For instance, previous studies have not found any association between sensitivity to TFS information and audiometric configuration (Hopkins and Moore, 2007; Strelcyk and Dau, 2009). This indicates that impairments to the processing of TFS information are relatively independent of hearing loss. Our results corroborate previous results as there was no association between sensitivity to TFS information at 0.5 kHz and audiometric thresholds. Age and sensitivity to TFS were associated with one another, but only weakly. In a recent study, however, Moore et al. (2012), found sensitivity to TFS worsen with age when assessing in a sample of 39 adults with ages ranging from 61 to 83 years (mean 69 years) with age-related hearing loss.

We found a number of self-report outcomes to be moderately associated with sensitivity to TFS information. For instance, prior to the receipt of a hearing aid, participants with good sensitivity to TFS information reported having better Spatial hearing (e.g., *Can you tell right away whether it is the person on your left or your right, without having to look?*) and Qualities of hearing (e.g., *Can you easily ignore other sounds when trying to listen to something?*) than participants with poor sensitivity to TFS on the SSQ-A. However, participants with poor sensitivity to TFS reported experiencing greater improvements on the Spatial and Qualities of hearing dimensions of the SSQ-B than the participants with good sensitivity to TFS at the 3-month follow-up, and Speech and Qualities of hearing at the 6-month follow-up. These results suggest that listeners with poor sensitivity to TFS may experience poorer spatial and qualities of hearing than listeners with good sensitivity to TFS prior to their hearing aid fitting, and therefore, experience by contrast greater hearing aid benefit as their initial score was lower and consequently had more “opportunities for improvement” than listeners with good sensitivity to TFS information. Given these differences in self-reported listening abilities and the relative independence of TFS sensitivity and audibility, it appears that an assessment of a patient's sensitivity to low-frequency binaural TFS information could prove useful in managing the expectations of patients who are due to receive a hearing aid. Differences in expectations could, at least partly, explain the observed patter of results in which patients with high sensitivity to TFS may have higher expectations, and consequently more difficult to fulfill, while patients with poor sensitivity to TFS may have lower expectations, easier to fulfill. Perhaps with better management options that take sensitivity to TFS information into account this advantage could be increased further. For instance, there is some evidence to suggest that choice of compression algorithm could be informed by knowledge of a patient's ability to process TFS information (Moore, 2008).

### Self-reported hearing aid outcomes

Good practice guidelines for adult audiology in the UK (Department of Health, 2007) recommend that patients receive a follow up visit sometime after provision of hearing aids (normally 8–12 weeks post-fitting) in which an assessment of patient outcomes should be undertaken. There are a number of methods available to monitor hearing-aid outcomes including subjective (self-report questionnaires) and objective measures of speech intelligibility (e.g., speech reception threshold). However, GHABP (Gatehouse, 1999) is the recommended outcome tool for assessing hearing-aid outcomes. In the current study, we employed three self-report questionnaires to monitor hearing-aid outcome, as previous research has shown that outcomes can vary markedly from one assessment tool to another (Humes, 1999; Lunner, 2003; Walden and Walden, 2004). Our results also revealed marked differences in outcome as measured on different outcome tools, and suggest that a multifaceted appraisal of hearing-aid outcome might be warranted.

We found that, while the GHABP pre-fitting dimensions (i.e., Handicap and Initial Disability) were highly associated with one another, they were not associated with age, severity of hearing loss or GHABP post-fitting outcomes. At the 3-month outcome assessment the four GHABP outcome dimensions (Usage, Benefit, Residual Disability, and Satisfaction) were strongly associated with one another, but again largely independent of age and severity of hearing loss. Moreover, there was a striking dichotomy between self-reports obtained on the pre-defined and self-nominated listening scenarios. The individual needs that may arise when measuring hearing aid benefit across different domains can be better captured when the hearing aid user is giving the opportunity to self-nominate specific scenarios. Those self-nominated scenarios may be very specific and only relevant to a single hearing aid user. While the GHABP is sensitive for capturing those meaningful and idiosyncratic listening difficulties, our results showed that those dimensions are not associated to TFS. These findings limit the efficacy of the GHABP as an outcome tool, at least when comparing group data, but highlight its sensitivity in characterizing patient's needs and therefore treatment improvement (e.g., managing expectations and hearing aid fittings). The SSQ-A and SSQ-B, on the other hand, showed high levels of consistency between pre- and post-fitting assessments, and were moderately correlated with GHABP pre-fitting dimensions; the SSQ questionnaires were also the only ones, in this study, to reveal subjective differences between listeners with good and poor sensitivity to TFS. It has been reported that the severity of hearing loss is associated with the amount of hearing aid benefit and satisfaction (Walden and Walden, 2004) or hearing aid usage (Bertoli et al., 2009) that patients report. We found that, the associations between self-report scores, age and hearing loss (4-frequency PTA at better ear) were generally weak. Outcome scores for Benefit and Satisfaction (GHABP), Speech, Spatial and Qualities (SSQ-B), and Satisfaction (IOI-HA) showed moderate associations with audiometric threshold, but only at low frequencies (0.25 and 0.5 kHz), and that this effect was stronger at the 6-month follow-up than at the 3-month follow-up. Results also showed that the severity of high-frequency loss was inversely associated with the Residual Participation Restrictions and the Impact on Others dimensions of the IOI-HA at the 6-month follow-up, indicating that those patients with the greatest levels of high-frequency hearing loss were least worried about the impact of their hearing loss on their daily lives and the lives of others. We also found that participant reports of hearing aid usage and benefit increased significantly over the 6-month follow-up period. However, neither outcome dimension increased significantly on a single outcome tool (Usage as measured on the IOI-HA increased during this period, but Usage on the GHABP did not; reports of Benefit on the GHABP increased over this period, but Benefit on the IOI-HA did not). Such variability in outcome highlights the differences in test sensitivity of the different methods, and the inherent limitations of restricting the clinical assessment of outcome to a single tool.

## Conclusion

In the current study, our hypothesis was that those patients with good sensitivity to TFS would have significantly better outcomes than those with poor sensitivity. Our results show that assessing sensitivity to TFS information could prove important to the management of the expectations of first-time hearing aid users. We found that, new hearing aid users with good sensitivity to TFS reported experiencing less debilitating hearing difficulties prior to provision of hearing aids, but also reported experiencing the least amount of improvement following provision of hearing aids compared to listeners with poor sensitivity to TFS. The TFS test employed in this study (TFS-LF: Hopkins and Moore, 2010a,b was designed to be quick, easy and clinically relevant). We have shown that even if a listener does not find the task easy, the test can be used to categorize listeners into two groups (good or poor sensitivity) that differ on subjective and objective measures of hearing aid outcome. These results provide further evidence about the role of TFS processing in understanding the difficulties faced by older listeners, and indicate that an assessment of sensitivity to TFS information could play a role in shaping the management of patients receiving hearing aids.

### Conflict of interest statement

The authors declare that the research was conducted in the absence of any commercial or financial relationships that could be construed as a potential conflict of interest.
